# Magnetic Resonance Imaging in the Clinical Evaluation of Lung Disorders: Current Status and Future Prospects

**DOI:** 10.1002/jmri.29802

**Published:** 2025-05-09

**Authors:** Linyu Wu, Chen Gao, Ting Wu, Ning Kong, Ziwei Zhang, Jie Li, Li Fan, Maosheng Xu

**Affiliations:** ^1^ Department of Radiology The First Affiliated Hospital of Zhejiang Chinese Medical University (Zhejiang Provincial Hospital of Chinese Medicine) Hangzhou China; ^2^ The First School of Clinical Medicine, Zhejiang Chinese Medical University Hangzhou China; ^3^ Department of Radiology Second Affiliated Hospital of Naval Medical University Shanghai China

**Keywords:** advancement, application, lung diseases, MRI

## Abstract

The low proton density and high signal decay rate of pulmonary tissue have previously hampered the application of magnetic resonance imaging (MRI) in the clinical evaluation of lung disorders. With the continuing technical advances in scanners, coils, pulse sequences, and image postprocessing, pulmonary MRI can provide structural and functional information with faster imaging speed and improved image quality, which has shown potential to be an alternative and complementary diagnostic method to chest computed tomography (CT). Compared with CT, MRI does not involve ionizing radiation, making it particularly suitable for pediatric patients, pregnant women, and individuals requiring longitudinal monitoring. This narrative review focuses on recent advances in techniques and clinical applications for pulmonary MRI in lung diseases, including lung parenchymal and pulmonary vascular diseases. Future developments, including artificial intelligence‐driven technological optimization and assisted diagnosis, hardware advancements, and clinical biomarkers validation, hold the potential to further enhance the clinical utility of pulmonary MRI.

**Evidence Level:**

5

**Technical Efficacy:**

Stage 2

## 1 Introduction

Lung diseases, including chronic respiratory diseases and lung cancer, are common abnormalities and one of the leading causes of death worldwide [1, 2]. Clinically, chest computed tomography (CT) has remained the main clinical technique for lung examination due to its speed, high resolution, and natural contrast effect in lung tissues [3]. Historically, pulmonary magnetic resonance imaging (MRI) has had limited clinical use due to multiple challenges related to rapid signal decay caused by the lung's low proton density, respiratory and cardiac motion sensitivity, and longer imaging acquisition times compared to CT [4]. However, recent advancements in pulse sequences, parallel imaging, and four‐dimensional (4D) free‐breathing acquisition techniques have significantly transformed pulmonary MRI [5, 6, 7].

**FIGURE 1:**
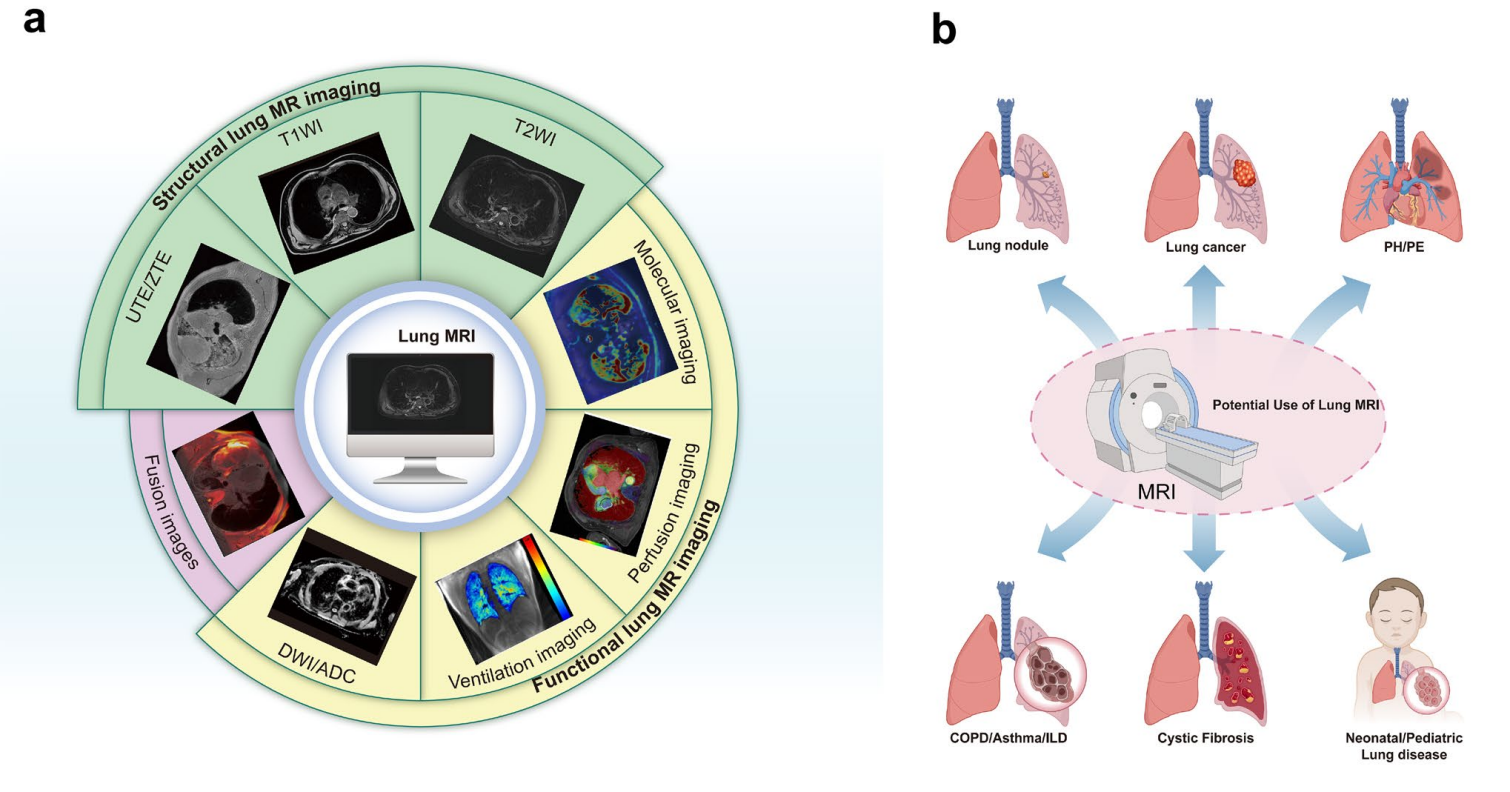
Lung MRI. (a) Composition of common pulmonary MR functional and structural imaging, fusion images such as zero echo time (ZTE) fused with MUSE DWI (*b* = 800 s/mm^2^); (b) Current potential uses of lung MRI in different specific clinical scenarios. ADC, apparent diffusion coefficient; COPD, chronic obstructive pulmonary disease; DWI, diffusion‐weighted MRI; ILD, interstitial lung disease; PE, pulmonary embolism; PH, pulmonary hypertension.

Pulmonary MRI remains one of the most challenging and attractive research fields. Despite numerous advances in lung MRI, its clinical utilization is still limited. In order to promote the appropriate clinical application of pulmonary MRI, the Fleischner Society published an expert consensus report in 2020 [3]. Pulmonary MRI offers unique possibilities for new biomarkers in lung diseases without ionizing radiation, with multiparameter imaging and quantitative regional lung functional information, which is especially vital for younger patients with chronic pulmonary diseases, children, pregnant women, and those requiring frequent long‐term follow‐up [5, 6, 7]. Current key clinical applications of lung MRI include lung cancer staging, lung nodule characterization, and the diagnosis and monitoring of pulmonary diseases such as cystic fibrosis (CF) and pulmonary hypertension (PH) [3]. To expand the role of pulmonary MRI in the clinical evaluation of lung disorders, this review provides an overview of state‐of‐the‐art technical advancements, mainly focusing on structural and functional imaging (Figure 1). This review also highlights the current clinical application for pulmonary MRI and offers an outlook on future directions.

## 2 Technical Innovations in Lung MRI

Recent advancements in MRI encompass enhanced pulse sequences, advanced multi‐coil parallel imaging, acceleration methods, and available postprocessing software [3] along with hardware development such as ultra‐low field (0.05 or 0.55 T) imaging [8, 9, 10] significantly reducing the limitations of lung MRI, making it more practicable. For pulmonary artery imaging, phase‐contrast MRI is a quantitative non‐contrast‐enhanced technique for evaluating pulsatile cardiovascular blood flow dynamics through time‐resolved velocity mapping, with two‐dimensional (2D) phase‐contrast imaging assessing unidirectional flow velocities and 4D phase‐contrast imaging extending this to volumetric, time‐resolved three‐directional velocity field quantification [11]‌. At the same time, the protein MRI contrast agents have shown potential applications in lung MRI. The effectiveness of protein MRI contrast agents, for example, chemical exchange saturation transfer (CEST) imaging, usually amide proton transfer weighted (APTw) imaging, has been verified in differentiating benign from malignant lesions of the lung or assessment of treatment response [12, 13].

The application of pulmonary MRI has expanded from structural imaging to functional imaging and even metabolic imaging, allowing separate reflection of anatomical, functional, and even metabolic information of the pulmonary lesions. Therefore, structural imaging, including ultra‐short echo time (UTE) and zero echo time (ZTE) sequences, the three common non‐contrast‐enhanced functional imaging is described as follows.

### 2.1 Improvements in Lung MRI Sequences of Structural Imaging

Structural imaging with lung MRI is always hampered by the rapid signal decay of limited protons of normal lung tissue and the very short T2* relaxation times of the lung [4]. A shorter echo time (TE) is paramount for preserving spatial resolution and signal fidelity in pulmonary MRI, with sequences such as UTE and ZTE providing near‐zero echo times. The utilization of UTE and ZTE sequences has significantly enhanced pulmonary structural imaging through effective signal capture from ultrashort‐T2 tissues and minimized signal decay in lung MRI.

UTE MRI minimizes rapid signal loss in short T2* lung tissues and improves lung structural imaging by achieving ultrashort echo times (UTE) of 0–200 μs through early free induction decay (FID) sampling post‐radio frequency excitation [14, 15]. In 1991, Bergin et al. first introduced three‐dimensional (3D) radial imaging techniques to achieve UTE for pulmonary MRI [16]. Subsequently, limited field‐of‐view excitation, variable readout gradients with eddy‐current corrections, and radial oversampling in 3D radial UTE protocols can significantly improve the technical quality of UTE lung images [14]. For 3D UTE MRI, two primary k‐space acquisition strategies are employed, and additional variations. The first utilizes radial sampling within a spherical geometry, commonly called the Pointwise Encoding Time Reduction with Radial Acquisition (PETRA) technique. The second employs cylindrical stacking using a stack‐of‐discs approach, known as the 3D‐UTE Stack‐of‐Spirals Volume‐Interpolated Breath‐hold Examination (3D‐USV) [17].

The structural difference between 2D and 3D UTE sequences arises from their technical implementations. Dual half‐pulse excitations with immediate readout to achieve ultrashort TE are used for the 2D UTE sequence, while the 3D UTE sequence usually employs hard‐pulse excitation combined with center‐out radial trajectories to optimize TE and spatial data capture, improving the quality of lung images [14]. Recent UTE sequences utilizing 3D radial trajectories have been refined for pulmonary imaging, achieving thoracic area coverage and near 1.0 mm isotropic spatial resolution comparable to standard or low‐dose chest CT examinations [14, 18, 19]‌.

The ZTE technique, another structural lung imaging method, achieves nearly zero TE by pre‐activating readout gradients before radio frequency excitation, enabling spatial encoding to synchronize with signal excitation [20]. The ZTE technique enables high‐resolution pulmonary structural imaging with superior signal‐to‐noise ratio and contrast‐to‐noise ratio in shorter scan times compared to UTE. However, one limitation is the flip angle, and allowed maximum receiver bandwidth [20]. Later, the refined adaptive ZTE k‐space trajectories (AZTEK) technique enables 3D dynamic ZTE lung imaging with retrospective gating by uniformly sampling the k‐space for any arbitrary respiratory motion gate, preserving static image quality, improving dynamic image quality, and ensuring continuous readout gradient transitions between spokes [21]. Nevertheless, developing UTE and ZTE sequences has promoted pulmonary structural imaging and has become a promising supplementary clinical imaging modality.

### 2.2 Innovations in Pulmonary Functional Imaging of MRI

Evaluating of respiratory mechanics, gas exchange, and pulmonary circulation is crucial for diagnosing and evaluating lung diseases. MRI is one of the techniques to assess the pathophysiology of pulmonary function on a regional level, including ventilation and perfusion imaging. For evaluating pulmonary ventilation, techniques included oxygen (O_2_)‐enhanced MRI, hyperpolarized gas MRI with helium 3 (^3^He) and xenon 129 (^129^Xe), fluorine‐ (^19^F‐) MRI, UTE ventilation MRI, and Fourier decomposition (FD) MRI [22, 23, 24, 25, 26, 27]. Dynamic contrast‐enhanced (DCE) MRI, arterial spin labeling (ASL), FD‐MRI, and related methods can also be utilized to assess pulmonary perfusion.

**FIGURE 2:**
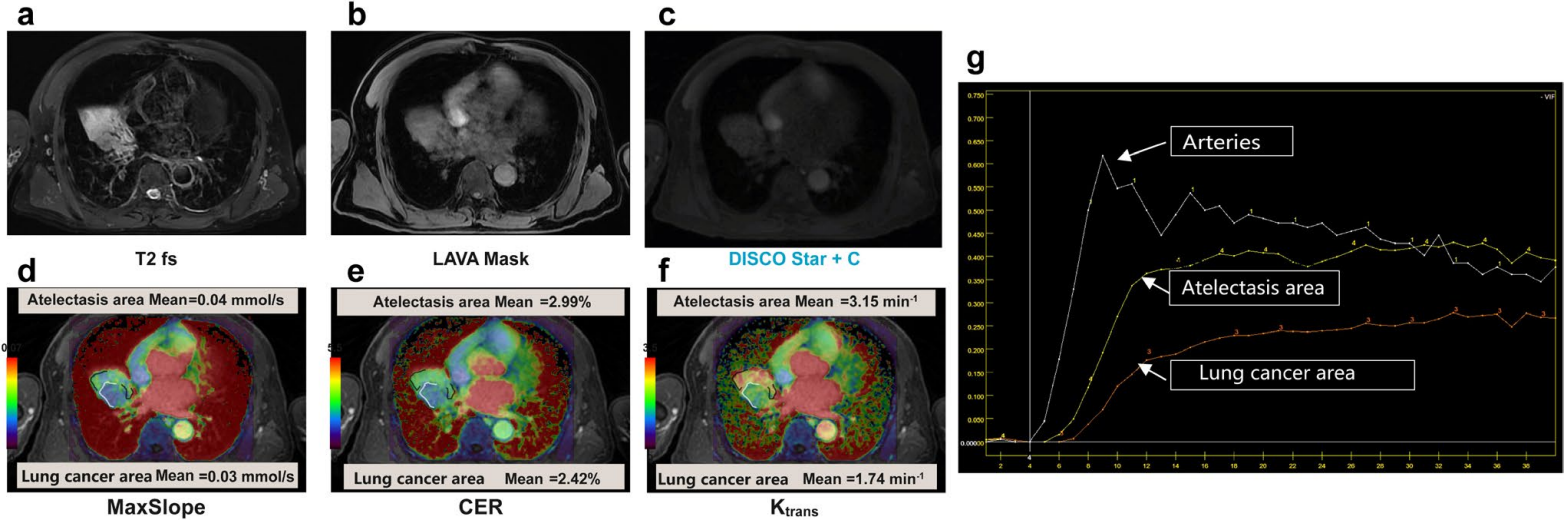
Male, 68 years old, right middle lobe lung cancer with right middle lobe lung atelectasis. (a) T2WI; (b) LAVA Mask; (c) DISCO Star + C; (d) maximum slope map, 0.03 mmol/s in the area of lung cancer, 0.04 mmol/s in the area of lung atelectasis; (e) contrast enhancement ratio map, 2.42% in the area of lung cancer, 2.99% in the area of lung atelectasis; (f) transfer constant map, 1.74 min^−1^ for lung cancer region and 3.15 min^−1^ for lung atelectasis region; and (g) dynamic enhancement curve.

While DCE‐MRI, especially with free‐breathing acquisition such as differential subsampling with Cartesian ordering (DISCO) Star using a gadolinium‐based contrast agent, is arguably the widely used and extensively studied perfusion technique for functional lung MRI [28] (Figure 2). The primary safety considerations of intravenously DCE‐MRI of gadolinium‐based contrast agents include risks of acute allergic‐like reactions, nephrogenic systemic fibrosis, gadolinium deposition, and symptoms associated with gadolinium exposure [29]. Therefore, non‐gadolinium‐based contrast‐enhanced functional lung MRI is a promising imaging technique that can provide important information for evaluating lung diseases without the risk of contrast agents. Three functional lung MRI approaches, including ventilation and perfusion imaging, are presented and discussed: inhaled‐gas ventilation MRI, ASL, FD, and related methods.

#### 2.2.1 Inhaled‐Gas Ventilation MRI

The commonly inhaled‐gas ventilation imaging methods for lung MRI mainly include O_2_‐enhanced MRI, hyperpolarized gas MRI with ^3^He and ^129^Xe, and fluorine‐ (^19^F‐) MRI [22, 23, 24, 25]. O_2_‐enhanced MRI quantifies regional oxygenation by comparing T1‐weighted signal changes between normoxic (21% O_2_) and hyperoxic (100% O_2_) conditions, with dynamic analysis of washin/washout rates of T1 relaxation time shortening caused by paramagnetic dissolved oxygen in lung tissue and blood [22, 30, 31]‌. Edelman et al. pioneered the O_2_‐enhanced MRI study for ventilation scanning using inhaled molecular oxygen as a contrast agent [22]. The O_2_‐enhanced MRI study by Jakob et al. demonstrated a statistically significant reduction in lung T1 values during 100% O_2_ inhalation compared to room air (*p* < 0.0001)‌ [30]. Although T1‐weighted oxygen‐enhanced MRI remains the predominant approach, T2*‐based quantification provides a more sensitive and physiologically direct ventilation measurement, with both mechanisms demonstrating comparable relative differences (about 10%–12.5%) in healthy volunteers following 100% oxygen administration [30, 32]. Therefore, a robust method for simultaneous quantifying T1 and T2* in the human lung during free breathing was proposed using UTE MRI, where T1 shortening reflects an increased amount of dissolved molecular oxygen in lung tissue, while T2* shortening indicates an elevated concentration of oxygen in alveolar gas [33]. The repeatability and feasibility of using 3D radial UTE oxygen‐enhanced MRI for functional imaging of asthma and CF have been established [34]. O_2_‐enhanced MRI is a relatively low‐cost and physiologically relevant imaging technique. However, it is limited to an compound assessment of ventilation, perfusion, and diffusion capacity [31, 35].

^19^F gas MR imaging capitalizes on the high gyromagnetic ratio of ^19^F and rapid T1 recovery (20 ms) of fluorinated gases to achieve an adequate signal‐to‐noise ratio with thermal polarization. At the same time, their chemical inertness enables safe, prolonged inhalation of normoxic ^19^F/O_2_ mixtures for quantitative regional lung ventilation mapping, even in slow gas‐exchange zones. Early studies on ^19^F gas MRI demonstrated the feasibility of breathing dynamics to be captured during a succession of short breath holds by rapid 3D spin density mapping (10‐s acquisition) in explanted human lungs using C_2_F_6_ and the feasibility of quantitative regional pulmonary pressure of oxygen using perfluorocarbon aerosols in vivo rat models [36, 37]. Subsequently, quantifying of regional lung ventilation using dynamic ^19^F gas washout MRI during free breathing is feasible at 1.5 T, even in obstructed lung segments [25]‌. However, ^19^F MRI requires exogenous ^19^F signals acquired via dedicated radiofrequency coils compared to O_2_‐enhanced MRI and exhibits a relatively low signal‐to‐noise ratio compared to hyperpolarized ^129^Xe gas MR imaging [25].

The gyromagnetic ratio of ^3^He is three times that of ^129^Xe, offering superior spatial resolution and signal‐to‐noise ratio, yet its scarcity and expense pose significant limitations for clinical applications. Compared to ^3^He, ^129^Xe gas has a larger natural gas inventory, making hyperpolarized ^129^Xe gas MRI more feasible and lower in cost for clinical studies. The intensity of the ^129^Xe MRI signal is directly proportional to its concentration after inhaling ^129^Xe gas. ^129^Xe MRI has a gaseous and dissolved phase, which includes ventilation imaging, diffusion‐weighted MRI (microstructure), and dissolved‐phase ^129^Xe MRI (gas exchange) [38]. Techniques via hyperpolarized ^129^Xe gas MRI imaging are sensitive to reflecting regional airway obstruction, changes in alveolar‐airspace size, and gas exchange at the alveolar‐capillary interface level [5, 39]. Because ^129^Xe imaging typically requires several breath‐holds with increased time and cost, a fast‐imaging sequence has been proposed that can acquire ^129^Xe MRI gas exchange and high‐quality ventilation images within a single breath‐hold of approximately 10 s [39].

#### 2.2.2 Unenhanced Perfusion Imaging: ASL

ASL is another noninvasive MRI approach without contrast agents based on extrinsically induced signal alterations by magnetically labeling arterial blood with radiofrequency pulses, generating a perfusion‐weighted difference image through the subtraction between control and labeled images [35, 40]. The perfusion‐weighted difference image in which the signal within a voxel corresponds to the volume of pulmonary arterial blood supplied during the preceding heart cycle, reflecting the perfusion information of the lung [35].

The initial application of ASL in pulmonary perfusion MR imaging was achieved by Hatabu et al. using an UTE turbo FLASH sequence with the signal targeting with alternating radiofrequency (STAR) technique [41]. This was subsequently followed by the development of the flow‐sensitive alternating inversion recovery (FAIR) technique and its enhanced version, FAIR with extra radiofrequency pulse (FAIRER) [42], offering more refined methods for capturing perfusion dynamics in the lung. Furthermore, single‐shot techniques like double inversion recovery (DIR) [43] have been proposed for pulmonary imaging to prevent artifacts and misregistration caused by differing respiratory levels in traditional ASL methods by completely suppressing stationary tissue. However, when the expiratory breath‐hold technique is applied in ASL methods, it results in reduced perfusion signal intensity within healthy pulmonary parenchyma, thereby diminishing contrast resolution between normal and pathological tissues. In the recent study by Othman et al., free‐breathing pseudo‐continuous ASL (PCASL) MRI with multiple repetitions provided excellent image quality and a good signal‐to‐noise ratio between disease and normal lung tissue in most cases [44]. Although ASL and related techniques provide a noninvasive and quantitative assessment of lung perfusion, challenges such as failing to show clots in the pulmonary arteries themselves and false‐positive results occurring in air trapping or emphysema regions persist in clinical settings [44].

#### 2.2.3 Unenhanced Ventilation and Perfusion Imaging: FD and Related Methods

In recent years, considerable effort has been made to develop MRI techniques of noninvasive, radiation‐free, and contrast agent‐free lung functional imaging that enable the assessment of lung ventilation and perfusion, are mainly based on intrinsic physiological signal oscillations of respiration and blood flow in the lung parenchyma [40]. FD MRI, developed over a decade ago, is one of the first widely explored and validated contrast‐agent‐free functional techniques, and simultaneous ventilation and perfusion measurement at the respiratory or cardiac cycle [45, 46, 47, 48]. Implementation on 1.5 T scanners requires an optimized multislice balanced steady‐state free‐precession (bSSFP) sequence in free‐breathing. Phase‐cycled bSSFP imaging for non‐contrast‐enhanced functional lung imaging exhibits enhanced resistance to magnetic field inhomogeneity‐induced artifacts while demonstrating notable improvements in ventilation map uniformity at 1.5 and 3 T [49]‌. This approach has been refined into several FD‐related techniques, including self‐gated non‐contrast‐enhanced functional lung (SENCEFUL) [50], matrix pencil decomposition MRI (MP MRI) [51, 52], and phase‐resolved functional lung (PREFUL) [27] techniques.

Compared to conventional FD, the PREFUL MRI technique can quantify dynamic pulmonary ventilation by analyzing MRI signal variations linked to proton density changes during air exchange and assess perfusion through phase‐sorted spoiled gradient echo sequences, which detect unsaturated spins in blood flow with higher temporal resolution across entire respiratory and cardiac cycles [27]. Time to peak (TTP), ventilation (V)/perfusion (Q) maps, and fractional ventilation flow‐volume loops can be calculated from PREFUL MRI, with specific parameters including regional fractional ventilation (RFV), perfusion quantification, ventilation defect percentage (VDP), perfusion defect percentage (QDP), and ventilation/perfusion match percentage (VQM) [27, 53]. Moreover, compared to FD and MP methods, the dynamic mode decomposition is employed to reliably derive functional maps for evaluating ventilation and perfusion, while mitigating amplitude fluctuations arising from variations in measurement count [54]‌.‌

**FIGURE 3:**
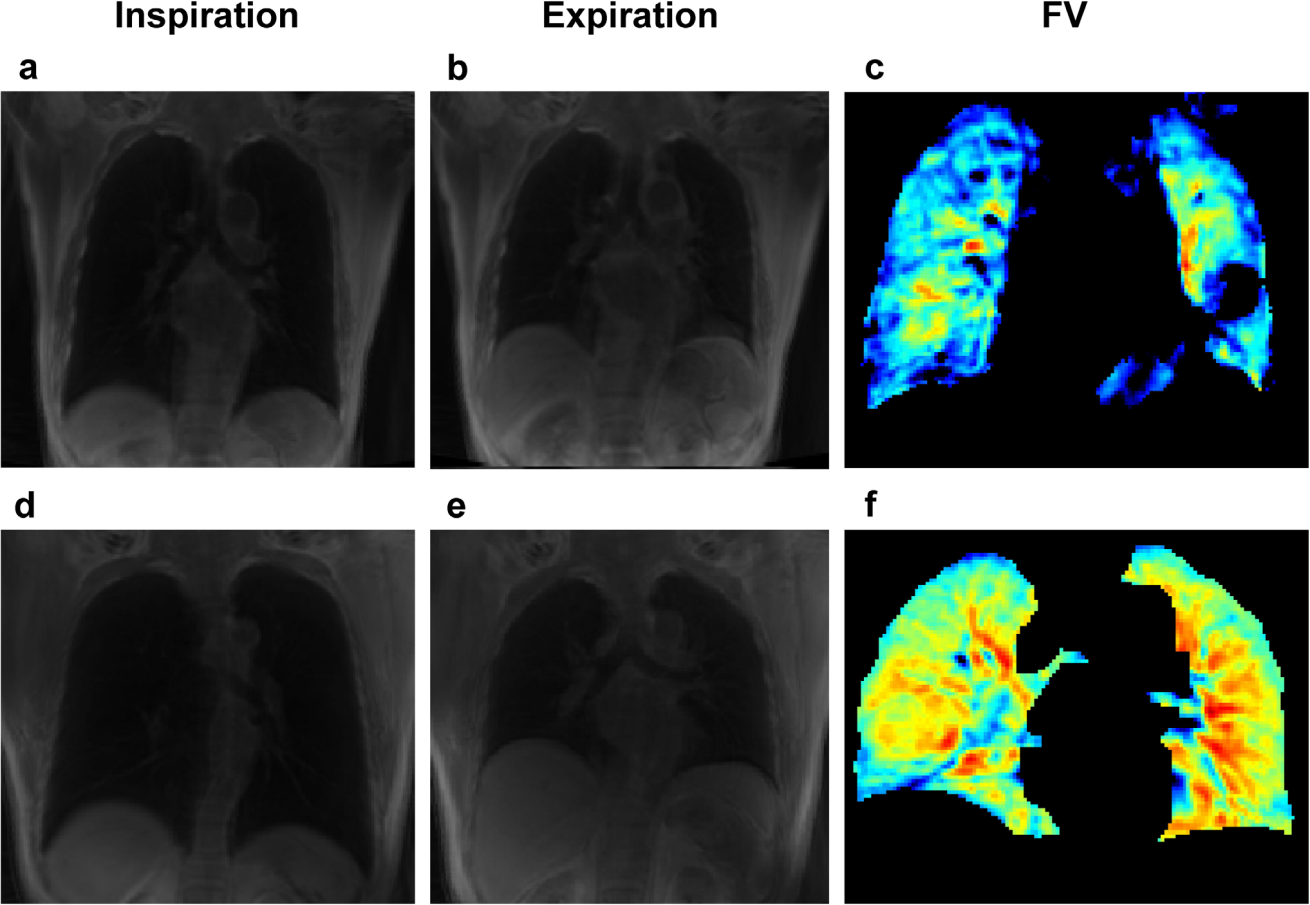
Upper row: Male, 74 years old, mass in the middle lobe of the right lung. (a) End‐inspiratory breath‐hold UTE; (b) end‐expiratory breath‐hold UTE; (c) ventilation maps at the lesion level, FV = 0.15. Lower row: Male, 48 years old, normal volunteer. (d) End‐inspiratory breath‐hold UTE; (e) end‐expiratory breath‐hold UTE; and (f) ventilation maps at the center level, left lung FV = 0.31, right lung FV = 0.32.

Non‐contrast enhanced functional imaging combined with morphologic imaging, such as UTE, aims to develop radiation‐free functional lung MRI (Figure 3). By analyzing the signal changes in the lungs during breathing cycles, technical approaches that seek ventilation imaging, such as UTE ventilation MRI [26, 55], can provide the benefits of an increased signal‐to‐noise ratio. 3D UTE MRI can quantitatively assess local ventilatory abnormalities, such as ventilation defect percentage, and lung morphologic changes, with an increased signal‐to‐noise ratio [26, 55]. Heidenreich et al. conduct functional lung MRI in patients with CF using a single breath‐hold 3D UTE sequence to create image‐based functional lung parameters to detect local ventilatory abnormalities, and monitor disease change [26, 55].

Additionally, further studies evaluated the reproducibility of non‐contrast‐enhanced multi‐breath‐hold or free‐breathing UTE functional lung MRI, and the results indicate that it provides highly reproducible ventilation imaging [56, 57]. Heidenreich et al. found that breath‐hold and self‐navigated 3D UTE sequences yield proton density‐weighted images of the lungs that are similar in image quality and both suitable for functional image analysis [58]. Though recent results from studies are promising, UTE ventilation MRI needs further verification in larger clinical samples.

Moreover, non‐contrast‐enhanced proton‐based MRIs, such as PREFUL and SENCEFUL, are promising for ventilation and perfusion in assessing the lung in free breathing. However, in contrast to UTE sequences, detailed morphological information is lacking. Therefore, recent efforts have been made to conduct SENCEFUL or PREFUL MRI using a free‐breathing 2D or 3D UTE acquisition to improve the signal‐to‐noise ratio for pulmonary ventilation and perfusion imaging [59, 60, 61, 62]. In 2019, Pereira et al. first developed a 3D‐UTE‐based SENCEFUL MRI to assess lung ventilation in free breathing, which is a robust method to assess both morphological and functional information of the lungs [59]. In addition, 2D or 3D UTE‐based PREFUL MRI in free breathing was also developed [60, 61]. This study compared PREFUL MRI using 3D UTE acquisition with other methods to evaluate ventilation and found that it showed good agreement with ^129^Xe‐MRI, conventional 2D multislice PREFUL MRI, and pulmonary function tests in CF patients [61]. In addition, a reconstruction pipeline for the 3D fermat‐looped orthogonally encoded trajectories (FLORET) UTE MRI was first proposed by Klimes et al., offering improved spatial resolution and strong correlation with ^129^Xe MRI and enabling dynamic ventilation quantification [62].

Recent studies demonstrate that free‐breathing ^1^H MRI (especially 3D UTE free‐breathing lung MRI, and free‐breathing 3D PREFUL MRI) exhibits correlation with hyperpolarized ^129^Xe MRI in quantifying ventilation abnormalities, particularly in capturing regional ventilation heterogeneity during free‐breathing conditions in common lung diseases [61, 63, 64]‌. However, most studies of contrast‐agent‐free functional MRI are based on a small sample in single‐center studies, which require additional prospective validation in clinical settings. In the future, emphasizing additional clinical prospective validation with established diagnostic methods, conducting multicenter studies on larger patient populations, and working towards standardizing the functional lung outcomes could be the focus of future research utilizing contrast‐agent‐free functional MRI.

## 3 Clinical Applications of MRI in Lung Disease Diagnosis

MRI is gradually being applied to the diagnosis and quantitative study of lung nodules, lung cancer, pulmonary parenchymal diseases, interstitial diseases, vascular diseases, CF, and other conditions (Figure 1) [3]. This section introduces the promising advances in the applications of pulmonary MRI in clinical lung disorders.

### 3.1 Lung Nodule and Lung Cancer

While CT is the reference for lung imaging in clinical practice, MRI plays a role in specific clinical scenarios, such as lung nodule detection, diagnosis, or cancer staging [18, 65, 66]. In earlier years, the performance of different MRI sequences in detecting pulmonary nodules needed improvement [67, 68]. For instance, the overall sensitivity of MRI sequences (T2‐TSE, T2‐SPIR, T2‐STIR, T2‐HASTE, T1‐VIBE, and T1‐out‐of‐phase) in detecting pulmonary nodules was 80.5%. Sensitivity varied based on nodule size, ranging from 57.1% to 87.5% for nodules ≤ 8 mm. However, this was accompanied by many false‐positive diagnoses [68]. In a high‐risk population, the overall sensitivity and specificity of MRI for lung nodule detection were only 48% (26/54) and 88% (29/33) compared to low‐dose CT [67].

**FIGURE 4:**
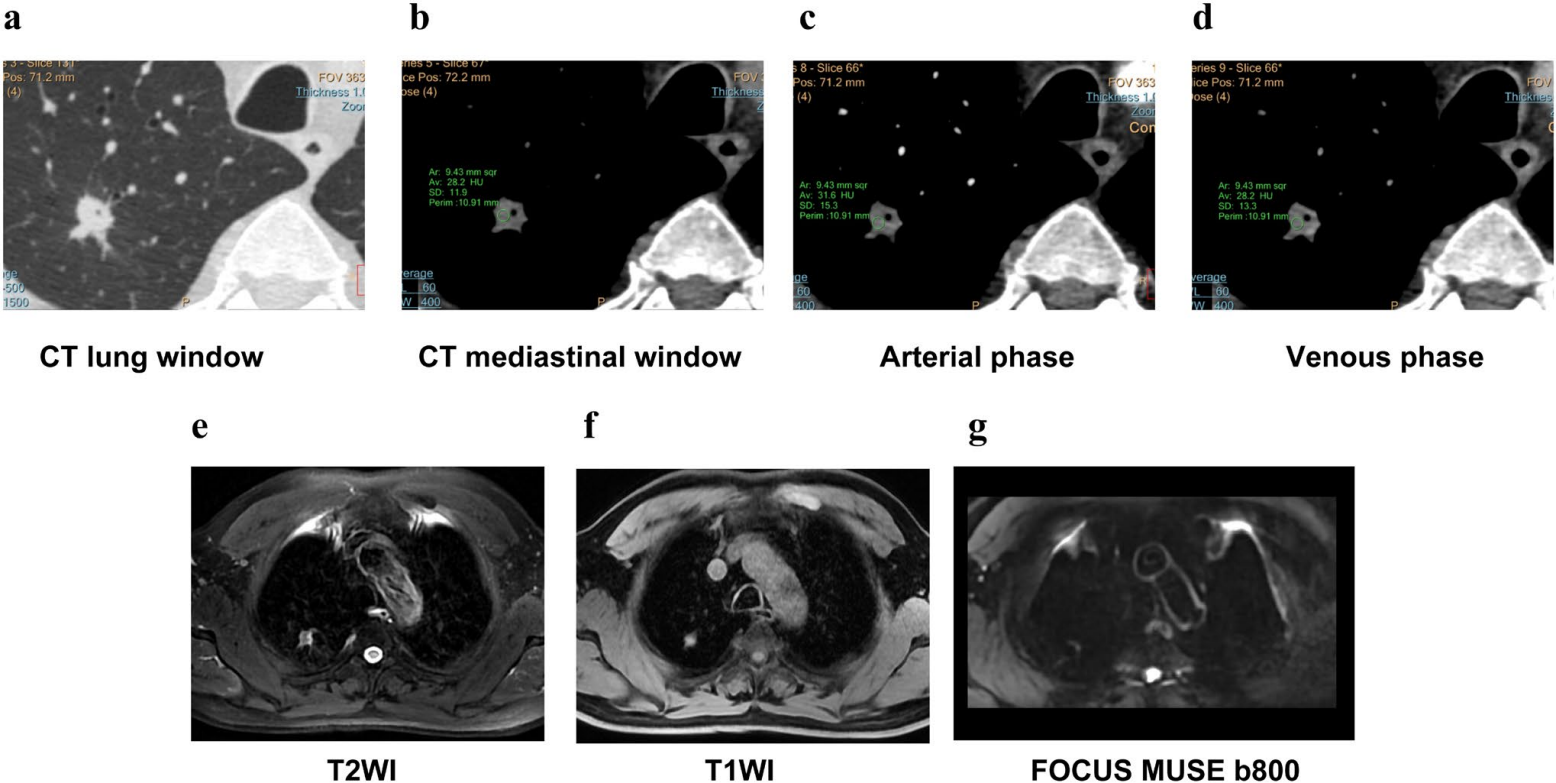
Male, 62 years old, solid nodule in the upper lobe of the right lung, approximately 24 mm in size, pathologically confirmed as tuberculosis. (a) CT lung window; (b) CT mediastinal window; (c) enhanced CT arterial phase mediastinal window; (d) enhanced CT venous phase mediastinal window; (e) T2WI; (f) T1WI; and (g) FOCUS MUSE DWI (*b* = 800 s/mm^2^). The lesion demonstrated no enhancement on contrast‐enhanced CT and exhibited low signal intensity on DWI sequences of MRI.

**FIGURE 5:**
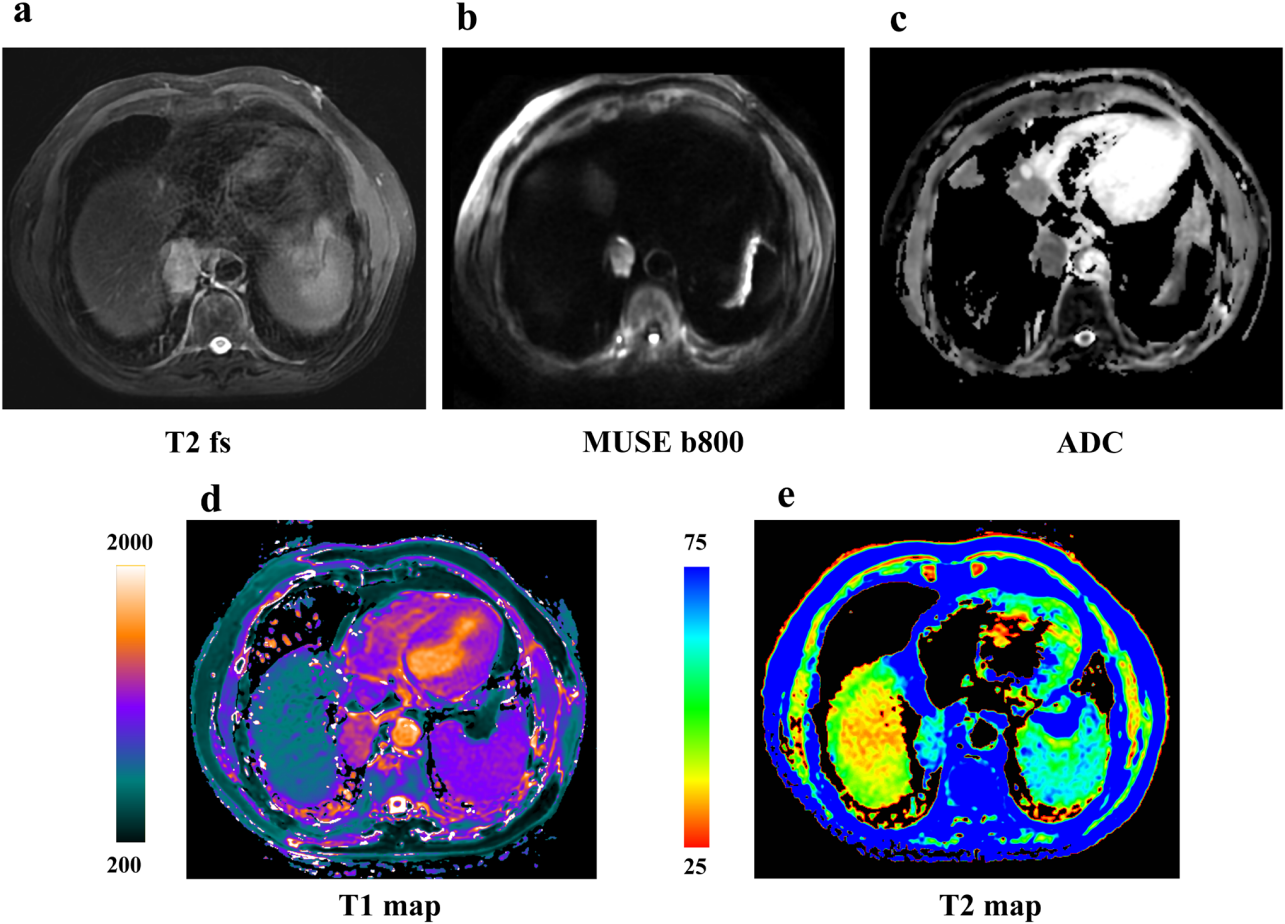
Male, 71 years old, soft tissue mass in the lower lobe of the right lung, about 43 mm in size, pathologically confirmed as squamous carcinoma. (a) T2WI; (b) MUSE DWI (*b* = 800 s/mm^2^); (c) MUSE ADC; (d) T1 mapping (ms); and (e) T2 mapping (ms).

**FIGURE 6:**
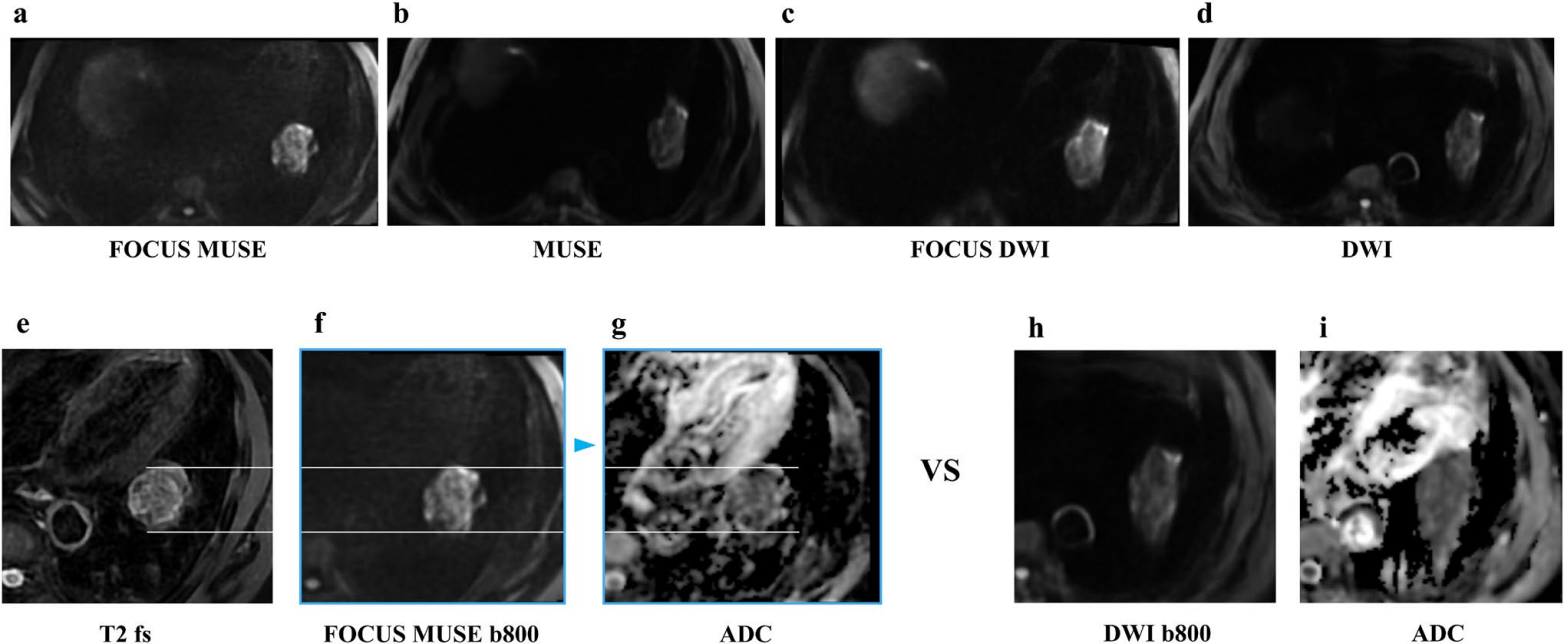
Male, 75 years old, soft tissue mass in the lower lobe of the left lung, approximately 41 mm in size. Pathology confirmed a malignant epithelial tumor with some neuroendocrine differentiation. (a) FOCUS MUSE DWI (*b* = 800 s/mm^2^); (b) MUSE DWI (b = 800 s/mm2); (c) FOCUS DWI (b = 800 s/mm^2^); (d) SS DWI (b = 800 s/mm2); (e) magnified lesion image on T2WI; (f) magnified lesion image on FOCUS MUSE b800; (g) magnified lesion image on FOCUS MUSE ADC; (h) magnified lesion image on DWI b800; and (i) magnified lesion image on DWI ADC.

**FIGURE 7:**
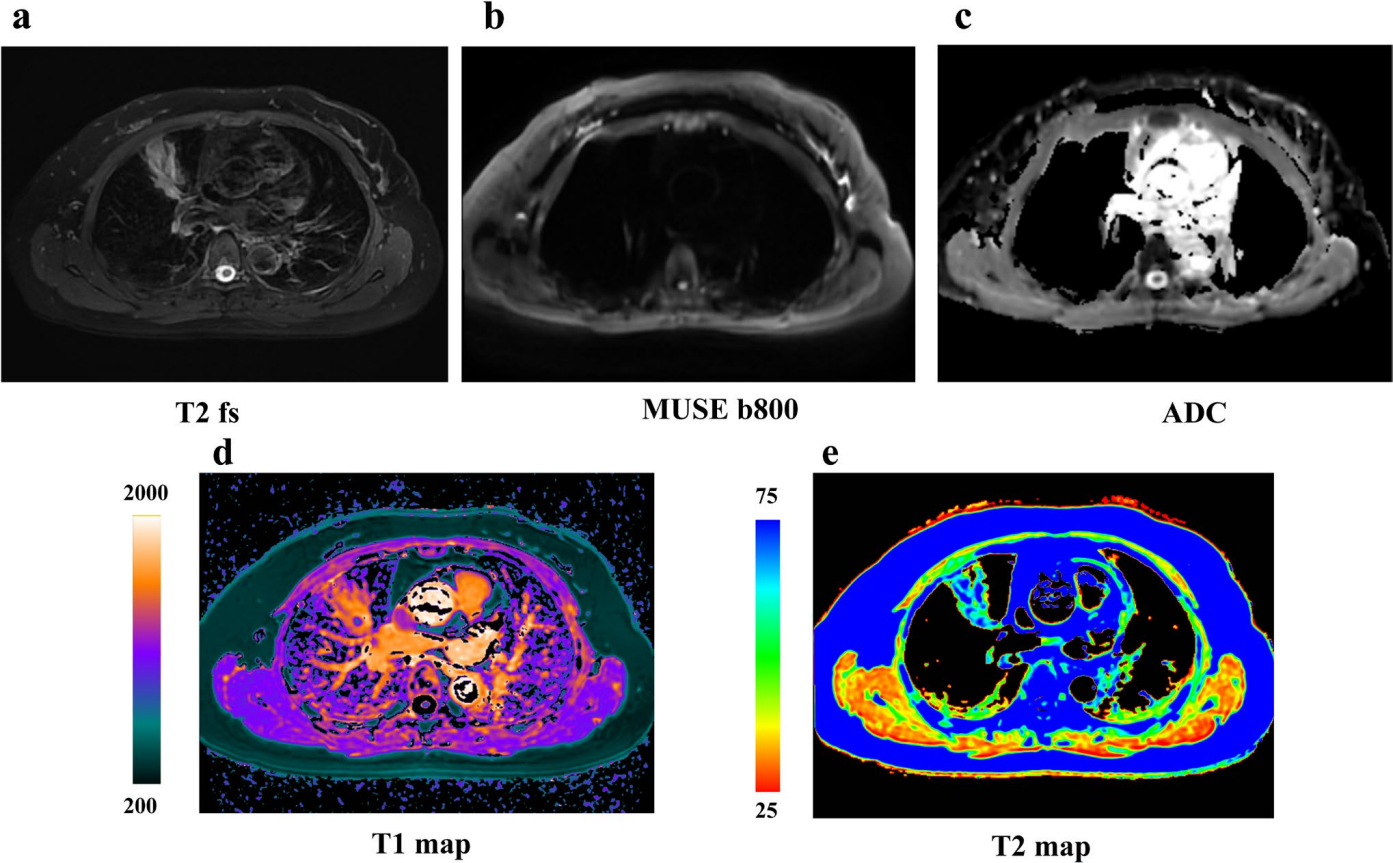
Female, 48 years old, solid nodule in the upper lobe of the right lung, approximately 20 mm in size, pathologically confirmed as an inflammatory lesion. (a) T2WI; (b) MUSE DWI (*b* = 800 s/mm^2^); (c) MUSE ADC; (d) T1 mapping (ms); and (e) T2 mapping (ms).

**FIGURE 8:**
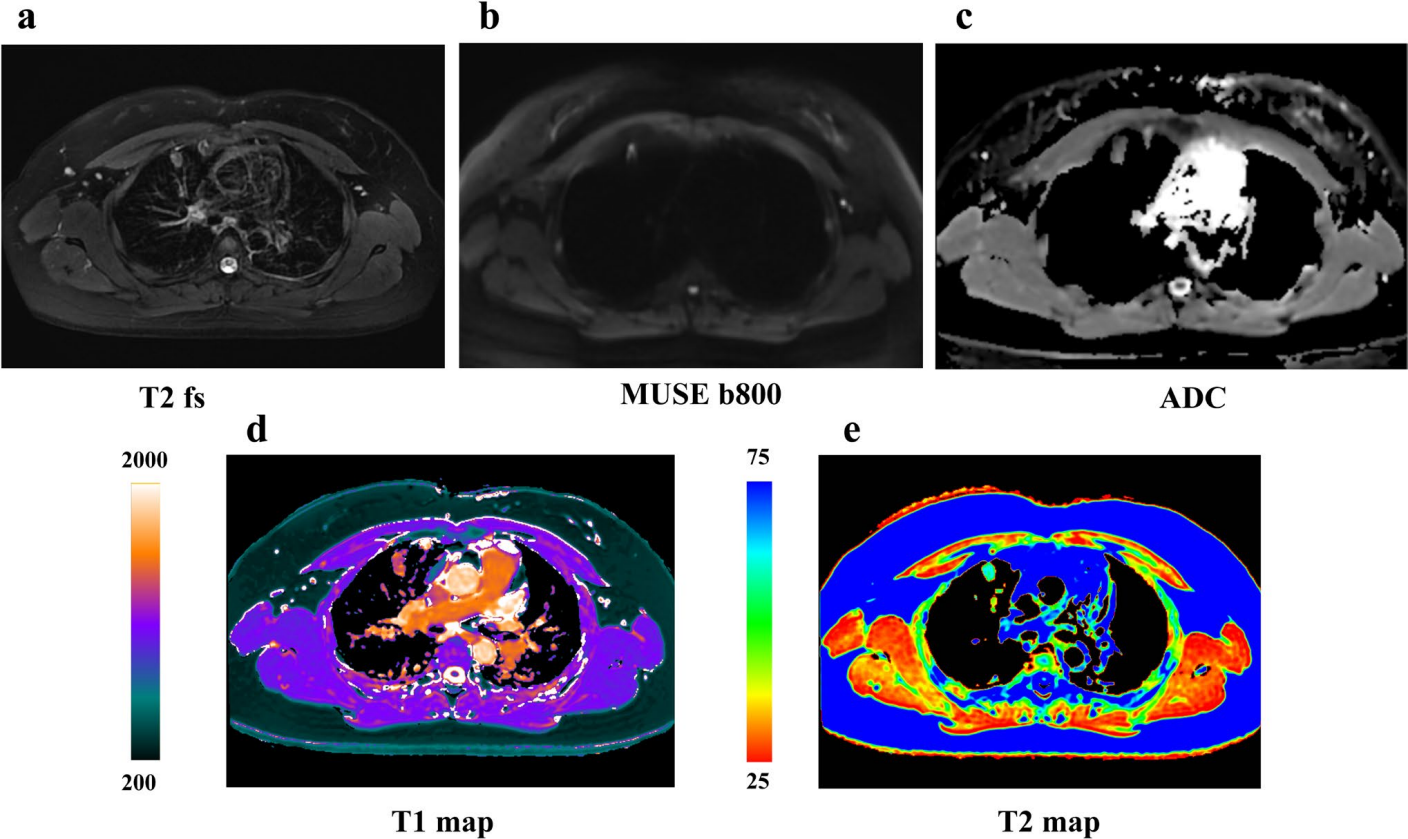
Female, 31 years old, solid nodule in the upper lobe of the right lung, approximately 15.6 mm in size, pathologically confirmed invasive lung adenocarcinoma with alveolar and papillary growths. (a) T2WI; (b) MUSE DWI (*b* = 800 s/mm^2^); (c) MUSE ADC; (d) T1 mapping (ms); and (e) T2 mapping (ms).

**FIGURE 9:**
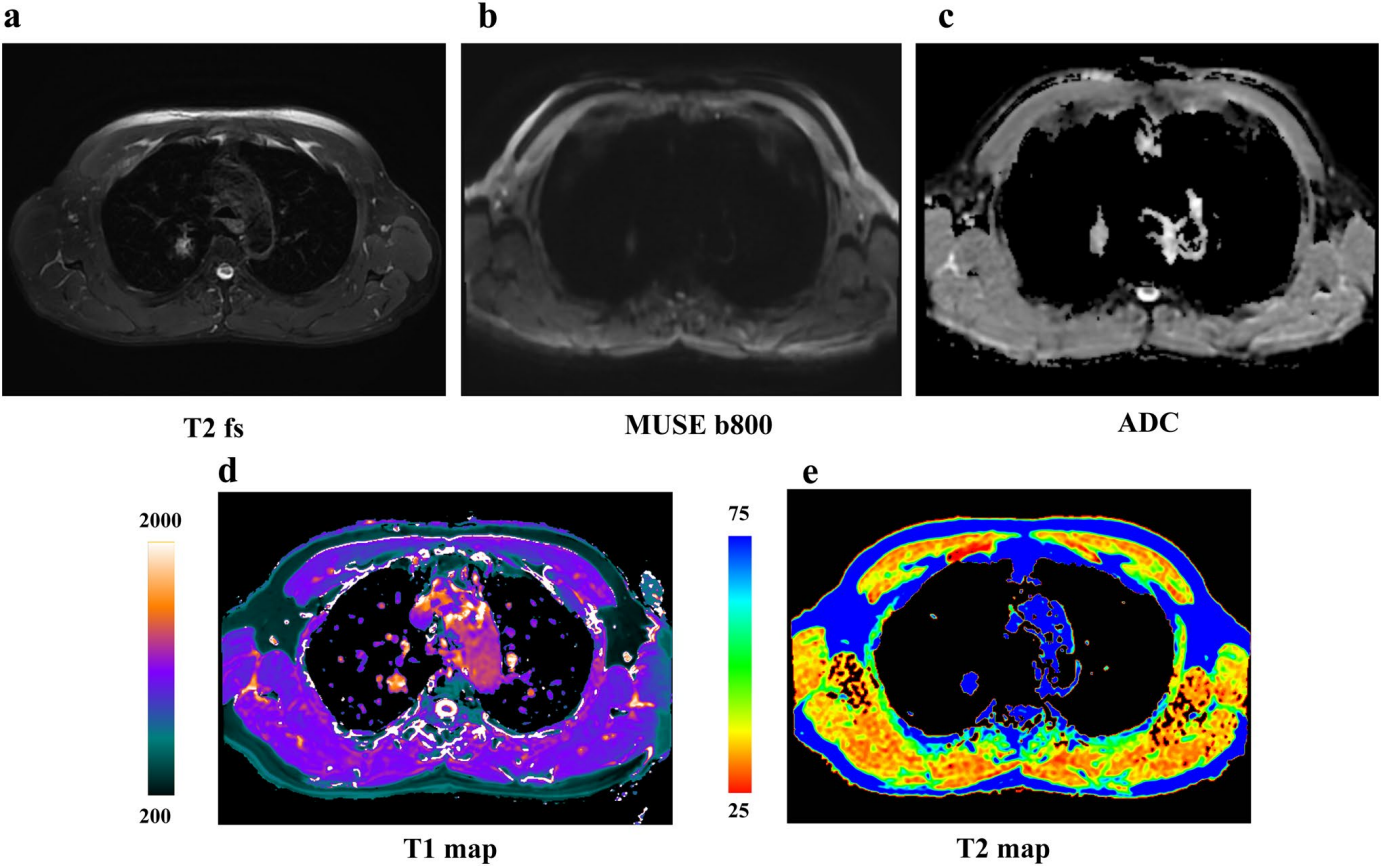
Male, 63 years old, nodule in the upper lobe of the right lung, approximately 13 mm in size, Pathology confirmed inflammatory granuloma. (a) T2WI; (b) MUSE DWI (*b* = 800 s/mm^2^); (c) MUSE ADC; (d) T1 mapping (ms); and (e) T2 mapping (ms).

**FIGURE 10:**
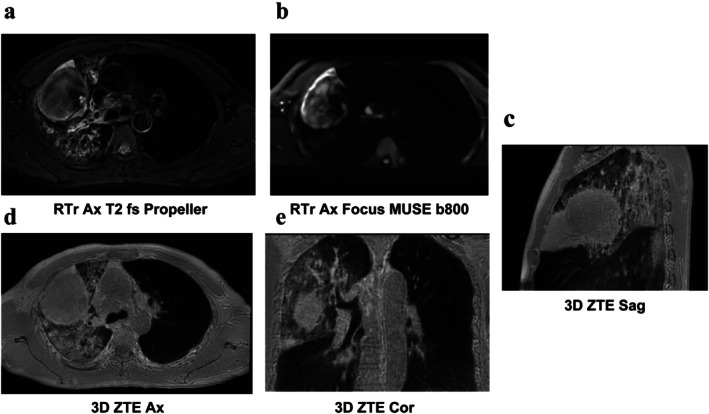
Male, 53 years old, lesion in the upper lobe of the right lung, compression atelectasis of the adjacent lung tissue, localized solid lesions, and enlarged lymph nodes visible in the mediastinum. (a) Respiratory‐triggered axial T2‐weighted fat‐saturated PROPELLER imaging‌‌, T2WI; (b) Respiratory‐triggered axial FOCUS MUSE DWI (*b* = 800 s/mm^2^); (c) 3D Sagittal ZTE; (d) 3D Axial ZTE; and (e) 3D Coronal ZTE.

**FIGURE 11:**
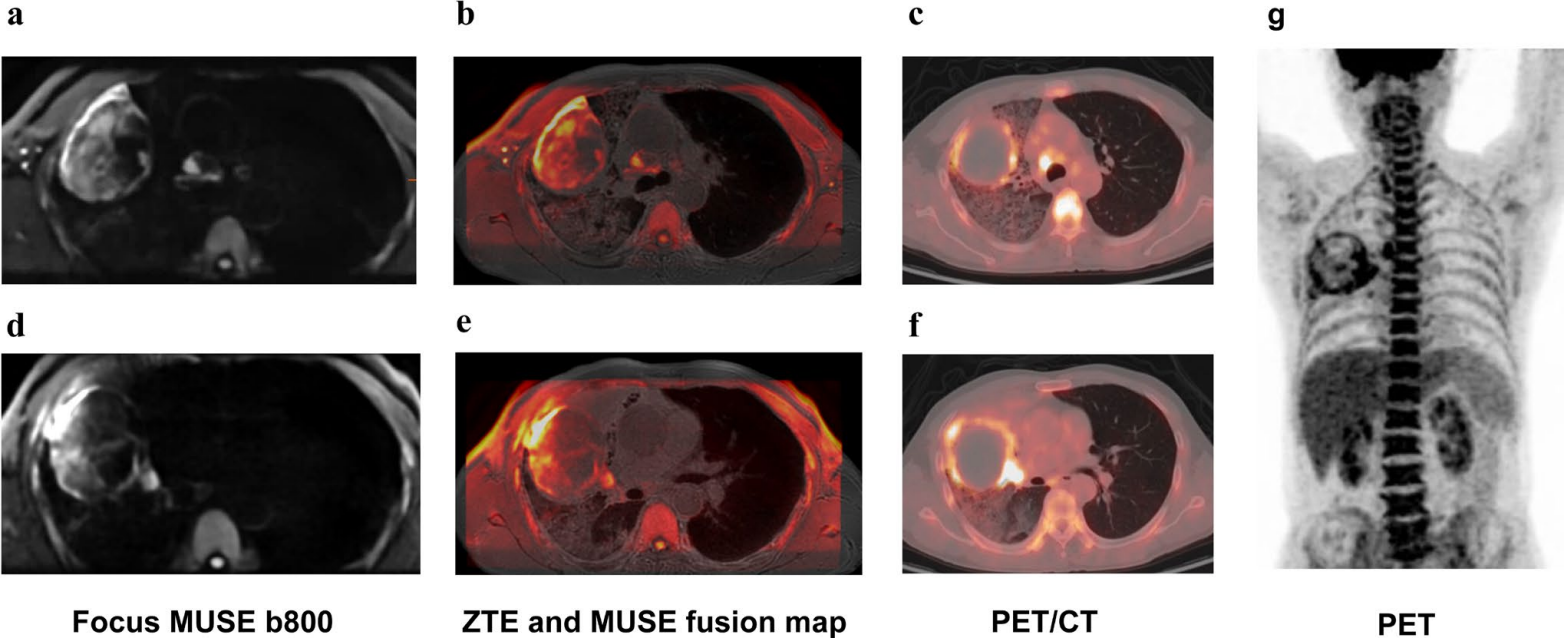
Male, 53 years old, lesion in the upper lobe of the right lung, compression atelectasis of the adjacent lung tissue, localized solid lesions, and enlarged lymph nodes visible in the mediastinum. (a, d) FOCUS MUSE DWI (*b* = 800); (b, e) ZTE and MUSE DWI fusion maps; (c, f, g ) 18F‐FDG PET/CT. The ZTE and MUSE DWI fusion maps show comparable performance to PET/CT imaging.

Moreover, diffusion‐weighted MRI (DWI), apparent diffusion coefficient (ADC) value, and DCE imaging are useful approaches for malignant and benign lesion differentiation and lung cancer staging [66, 69] (Figures 4, 5, 6, 7, 8, 9). The ADC value can provide a quantitative parameter to avoid the false positives in DWI caused by the T2 shine‐through effect when differentiating between malignant and benign pulmonary nodules or masses with a pooled sensitivity and specificity of 83% (95% CI: 75%, 89%) and 91% (95% CI: 80%, 96%), thereby providing substantial diagnostic value in clinical practice [66] (Figures 5 and 7, 8, 9). In a meta‐analysis evaluating the diagnostic performance of DWI for indeterminate pulmonary lesions, the optimal ADC cutoff values for differentiating malignant from benign pulmonary nodules/masses ranged from 0.4 to 1.78 × 10^−3^ mm^2^/s, with a median value of 1.15 × 10^−3^ mm^2^/s using *b* values of 500–1000 s/mm^2^ across 12 included studies [66]. While DWI of the lung encounters challenges, including susceptibility artifacts, motion‐related artifacts, and geometric distortion, field of view optimized and constrained undistorted multiplexed sensitivity encoding (FOCUS MUSE) DWI exhibits improved image quality and reduced deformation compared to multiplexed sensitivity encoding (MUSE), field of view optimized and constrained undistorted multiplexed sensitivity encoding (FOCUS), and single‐shot echo‐planar (SS) DWI in lung lesions (Figure 6). DWI has comparable diagnostic ability to that of ^18^F‐FDG PET/CT in N staging (Figures 10 and 11), and computed DWI with a *b* value at 600 s/mm^2^ may have the potential to improve differentiation of metastatic from nonmetastatic lymph nodes as compared with DWI and PET/CT [70].

**FIGURE 12:**
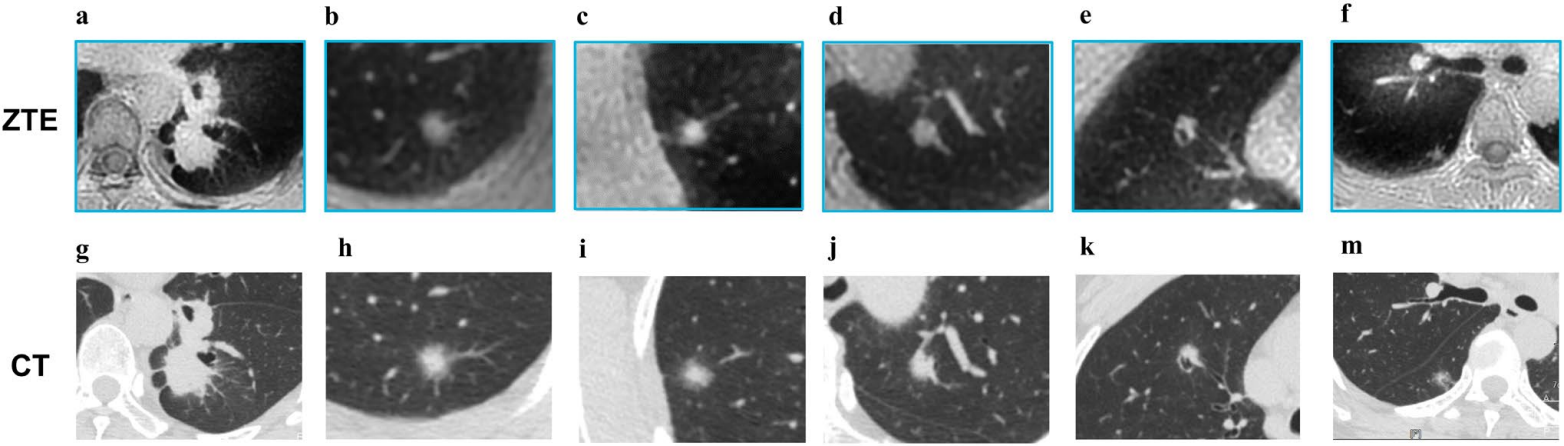
Female, 49 years old, multiple nodules in both lungs, dorsal roundish mass in the upper lobe of the left lung with clear borders and hairy edges, with lobulation, tracheal truncation, and cavity formation. (a–f) ZTE shows multiple nodules; (g–m) CT images correspond to ZTE.

**FIGURE 13:**
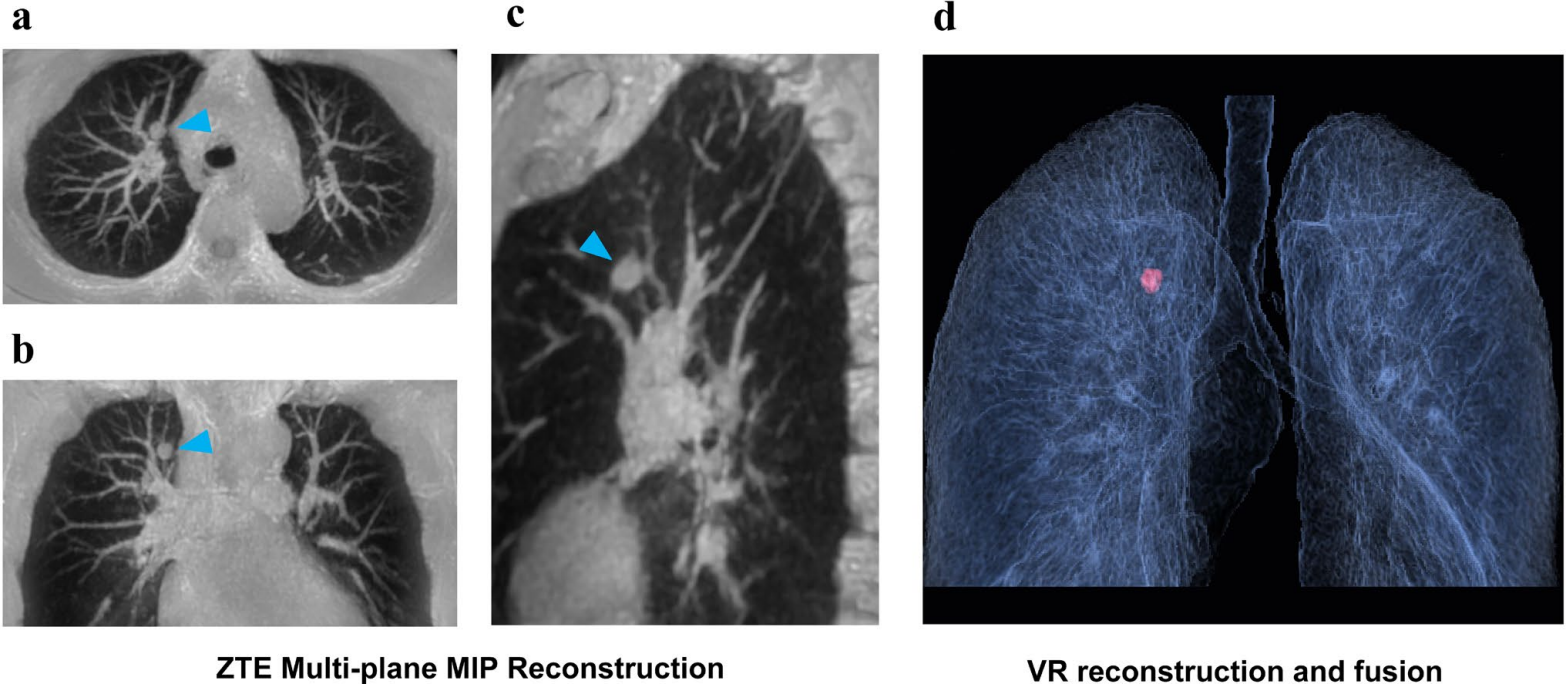
Male, 56 years old, small rounded nodule at the tip of the upper lobe of the right lung. (a) ZTE multiplanar MIP reconstruction of the transverse section; (b) ZTE multiplanar MIP reconstruction of the coronal section; (c) ZTE multiplanar MIP reconstruction of the sagittal section; and (d) VR reconstruction and fusion of ZTE images.

**FIGURE 14:**
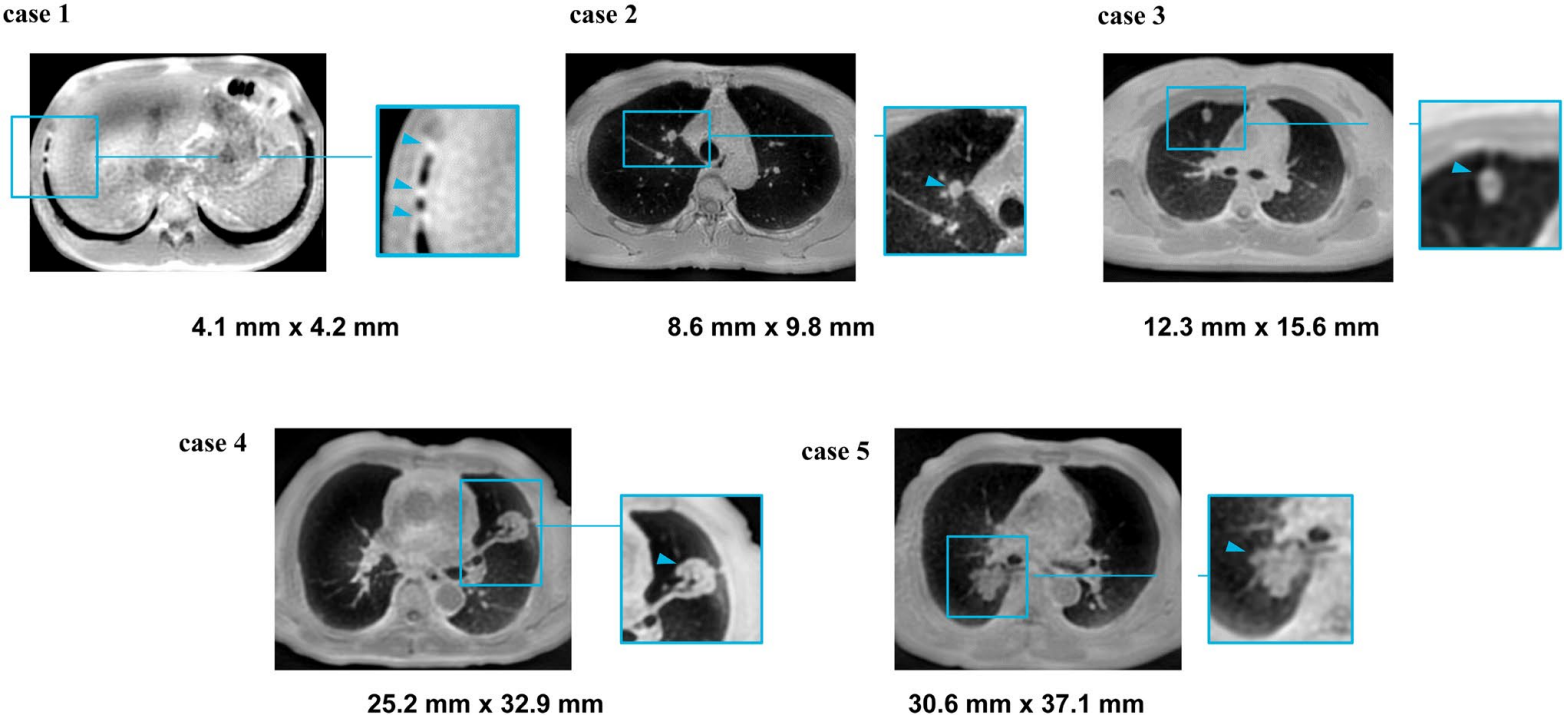
Case 1: Male, 32 years old, a small nodule in the lower lobe of the right lung, lesion size 4.1 mm × 4.2 mm. Case 2: Male, 56 years old, small nodule at the tip of the upper lobe of the right lung 8.6 mm × 9.8 mm. Case 3: Female, 31 years old, small nodule in the anterior segment of the upper lobe of the right lung 12.3 mm × 15.6 mm. Case 4: Male, 74 years old, left lung upper lobe mass 25.2 mm × 32.9 mm. Case 5: Male, 75 years old, right lung lower lobe mass 30.6 mm × 37.1 mm.

During the last several years, the emergence of pulmonary thin‐section MRI using UTE or ZTE sequences has increased the potential clinical utility and is a promising tool of pulmonary MRI in detecting and characterizing lung nodules for comparison with CT imaging [18, 19, 65, 71, 72, 73, 74] (Figures 12, 13, 14). Table 1 shows reported lung nodule detection performances of emerging UTE or ZTE sequences compared to standard‐dose CT [18, 19, 72, 73, 74]. 3D GRE sequences with UTE enabled a detection rate of 71.8% to 93.0% for all nodules and nearly 100% for nodules larger than 8 mm [18, 19, 72, 73, 74].

**TABLE 1:** Summary of detection performance by ultrashort echo time (UTE) and zero echo time (ZTE) of lung magnetic resonance imaging (MRI) for lung nodules and lung masses.

Author, year	Reference Standard	Slice thickness (CT)	Manufacturer (MRI)	Sequence of MRI	Slice thickness (MRI)	Compared the examine	Sensitivity (different nodule type)	Sensitivity (≤ 4 mm)	Sensitivity (> 4 mm)	Sensitivity (> 8 mm)
[72] Wang et al., 2025	Standard‐dose CT	1.5 mm	GE Healthcare	ZTE	1.4 mm	n/a	All nodules: 85.7% (54/63; SSN: 77.1% (27/35); SN: 96.4% (27/28)	n/a	n/a	n/a
[73] Sanchez et al., 2023	Standard‐dose CT	1.25 to 3 mm	Siemens Healthcare	UTE	2.5 mm	VIBE; HASTE	All nodules: 71.8% (107/149); SSN: 73.5% (36/49); SN: 71% (71/100)	≤ 4 mm: 31.9% (15/47)	> 4 mm: 90.2% (92/102)	n/a
[19] Ohno et al., 2022	Standard‐dose CT	1 mm	Canon Medical Systems	UTE	1 mm	Standard‐dose CT and low‐dose CT	All nodules: 87.9% (943/1073)	n/a	n/a	n/a
[18] Ohno et al., 2017	Standard‐dose CT	1 mm	Toshiba Medical Systems	UTE	1 mm	Standard‐dose CT and reduced‐dose CT	All nodules: 93.0% (226/243)	n/a	4–6 mm: 74.1% (43/58); 6–8 mm: 94.3% (33/35)	> 8 mm: 100% (150/150)
[74] Burris et al., 2016	Standard‐dose CT^a^	2.5 mm	GE Healthcare	UTE^b^	1.25 mm	Dual‐Echo GRE	All nodules: 73% (60/82)	2–4 mm: 17% (2/12)	4–6 mm: 71% (20/28); 6–8 mm: 83% (15/18)	> 8 mm: 95.8% (23/24)

Abbreviations: HASTE, half Fourier single‐shot turbo spin‐echo; n/a, not applicable; SN, solid nodule; SSN, subsolid nodule; VIBE, volumetric interpolated breath‐hold examination.

^a^ From PET/CT.

^b^ From PET/MR.

Other studies have also evaluated the potential of UTE or ZTE sequence for precise lung nodule morphological characterization, differentiation of lung lesions, and image quality [20, 65, 71]. Although UTE lung imaging generally underestimated the lung nodules' long axial diameter measurements by about 1–2 mm, pulmonary thin‐section MRI using UTE sequence was useful for detecting pulmonary nodules, assessing nodule type, and showing strong inter‐reader agreement compared to CT in evaluating lung nodule morphology [18, 71]. A similar performance is also observed in ZTE for lung nodules or masses, as shown in Figure 14.

Moreover, molecular MRI using protein MRI contrast agents, such as APTw imaging, is one subset of CEST imaging, which is useful for differentiating malignant from benign lesions [12, 13]. Magnetization transfer ratio asymmetry at 3.5 ppm of APTw imaging was significantly higher for malignant tumors (mean ± standard deviation, 3.56% ± 3.01%) than benign lesions [12]. The diagnostic performance of APTw imaging is comparable to DWI and 18F‐FDG PET/CT [13].

### 3.2 PH and Pulmonary Embolism (PE)

PH and PE are common pulmonary vascular diseases. Various imaging modalities, including conventional CT, dual‐energy CT, and advanced MRI techniques, provide imaging biomarkers in diagnosing, risk stratification, and predictive value of pulmonary vascular diseases like PH and PE [75, 76]. Advanced MRI techniques, such as DCE lung perfusion MRI and phase contrast flow MRI, using radiation‐ and gadolinium‐free MRI agents, can be promising in evaluating this pulmonary vascular disease [11, 75, 77].

DCE lung perfusion MRI can provide a detailed structural assessment of pulmonary vessels and functional information on pulmonary perfusion for chronic thromboembolic PH. It has been reported that DCE lung perfusion MRI can achieve a similar sensitivity and specificity for single‐photon emission CT screening for chronic thromboembolic PH [75]. Moreover, a recent study showed that PREFUL MRI is an alternative technique for evaluating regional ventilation and perfusion for diagnosing chronic thromboembolic PH [76].

Other advanced MRI techniques, especially phase contrast techniques, including routine 2D and 4D flow MRI, produce phase shifts in nonstationary protons to show moving fluid and are deemed the potential choice for early diagnosis and characterization of PH [11]. Novel emerging 4D flow MRI improved the spatial and temporal resolution, allowing the assessment of local flow, vorticity, and kinetic energy to track the vortex and estimate PH [78, 79]. Pulmonary artery stiffness and mean pulmonary arterial pressure can be evaluated by parameters generated from phase‐contrast MRI sequences, such as vortical blood flow, blood velocities, wall shear stress, and right ventricular diastolic dysfunction [78, 79].

In routine clinical practice, contrast‐enhanced CT angiography has become the first‐choice test for patients with suspected acute PE [80], replacing magnetic resonance angiography. Although promising, magnetic resonance angiography is not used in clinical practice due to its low sensitivity and lack of speed in an emergency [81]. In a recent study, the free‐breathing PCASL MRI technique can identify abnormal lung perfusion caused by acute PE with 92% sensitivity and 95% specificity and serves as a contrast material‐free alternative to CT pulmonary angiography for certain patients. However, it cannot visualize thrombi and may produce false positives in emphysema or air‐trapping areas [44]. A recent study has explored the applicability of ferumoxytol‐enhanced magnetic resonance angiography to assess PE in pregnancy and demonstrated that ferumoxytol‐enhanced magnetic resonance angiography could be a radiation‐ and gadolinium‐free alternative [77].

### 3.3 Chronic Obstructive Pulmonary Disease (COPD), Asthma, and Interstitial Lung Disease (ILD)

Lung diseases such as COPD, asthma, and ILD are very common chronic respiratory illnesses in the world that may affect pulmonary function [1, 2]. Aside from evaluating morphology and structure, lung function's regional and spatial information can be quantified by pulmonary functional imaging, including CT, MRI, and nuclear medicine techniques [7, 82]. At the same time, conventional pulmonary function tests or anatomy imaging cannot provide spatial localization information and microstructural quantitative data, which is important for longitudinal assessment of chronic respiratory illness [82]. According to the Fleischner Society guidelines, further research into lung MRI applications for COPD, asthma, and ILD is warranted, focusing on preclinical and patient studies. These areas represent key developments that require more investigation to advance the field [3].

Pulmonary ventilation can be evaluated by several MRI techniques, e.g., hyperpolarized gas MRI (^129^Xe, ^3^He), ^19^F‐MRI, O_2_‐enhanced MRI, and PREFUL (Table 2) [23, 25, 26, 31, 83, 84, 85, 86, 87, 88, 89, 90, 91, 92]. COPD, asthma, and ILD present with a wide range of phenotypes. The hyperpolarized gas MRI technique plays a significant role in pulmonary imaging, offering functional assessments and the ability to quantify regional changes in chronic respiratory diseases (Table 2) [23, 83, 84, 85, 86, 87, 88, 89, 90, 91]. This imaging technology has evolved to include static and dynamic ventilation imaging, oxygen–pressure mapping, ^129^Xe dissolved‐phase imaging, and chemical shift saturation recovery spectroscopy, making it applicable to a range of lung disorders such as COPD, asthma, and ILD (Table 2) [23, 83, 84, 85, 86, 87, 88, 89, 90, 91].

**TABLE 2:** Ventilation of magnetic resonance imaging (MRI) techniques in common chronic respiratory diseases.

Imaging methods	Nucleus of gas	Main diseases application	Metrics
Static ventilation MRI imaging	^3^He, ^129^Xe	COPD [23]; Asthma [83]	VDP
Multibreath MRI imaging	^3^He, ^129^Xe	CF [84]; COPD [85]	VDP; ventilation heterogeneity index; regional TV; FRC; FV; f_RBC_; f_Mem_; f_RBC:Men_
Hyperpolarized gas DWI or ADC mapping	^3^He, ^129^Xe	COPD [86]; Asthma and CF [87]; IPF [88]	ADC value
Dissolved‐phase imaging or CSSR MR spectroscopy	^129^Xe	COPD [89]; Asthma [90]; IPF [91]	VDP; ventilation percent; RBC‐to‐TP ratio; the RBC‐to‐GP ratio; RBC‐to‐B ratio
^19^F lung MRI	^19^F	COPD [25]	VDP; washout time; number of breaths; FV
Oxygen‐enhanced MRI	Oxygen	COPD [31]	ΔT_1_
FD‐MRI	n/a	COPD [92]	FD‐FV
UTE ventilation MRI	n/a	CF [26]	FV; UTE‐measured lung density and residual volume

Abbreviations: ADC, apparent diffusion coefficient; CF, cystic fibrosis; COPD, chronic obstructive pulmonary disease; CSSR, chemical shift saturation recovery; DWI, diffusion‐weighted imaging; FD, fourier decomposition; f_RBC_, f_Mem_, f_RBC:Mem_, efficiency of gas exchange with red blood cells (f_RBC_) and lung tissue (f_Mem_) and exchange between the red blood cells and tissue compartments (f_RBC:Mem_); FRC: functional residual capacity; FV: fractional ventilation; IPF, interstitial pulmonary fibrosis; n/a, not applicable; RBC‐to‐B, red blood cell to barrier; RBC‐to‐GP, red blood cell to gas phase; RBC‐to‐TP, red blood cell to tissue‐plasma ratios; ΔT_1_, relaxation time change between pre‐ and postoxygen inhalation; TV, tidal volume; UTE, ultrashort echo time; VDP, ventilation defect percentage.

Hyperpolarized ^3^He MRI has advanced from early studies on emphysema and lung cancer in mice [93] to in vivo lung morphometry measurements in COPD patients [94]. The use of parametric response mapping to quantify markers of emphysema and small airway disease has also been reported [95]. A significant breakthrough in asthma research using MRI involved mapping ventilation heterogeneity with hyperpolarized gas MRI, revealing a T2‐high phenotype in asthma patients [96, 97]. In studies of hyperpolarized gas MRI for ILD, biomarkers from ^129^Xe ventilation and oxygen‐enhanced MRI could not distinguish between ILD subtypes [98]. However, recent findings show that parameters like the apparent diffusion coefficient and mean diffusive length scale, derived from ^3^He diffusion‐weighted MRI, offer a noninvasive, quantitative assessment of microstructural acinar changes in ILD [88]. Otherwise, 3D‐ZTE lung MRI provides a reliable, radiation‐free alternative to CT for assessing fibrosis and ground‐glass opacities of ILD in systemic sclerosis patients [99].

### 3.4 CF

CF is an autosomal recessive genetic disease that affects at least 100,000 people worldwide and is most diagnosed in Caucasians [100, 101, 102]. Classical CF is a complex, multi‐organ disease that presents in the lung as mucus retention, chronic pulmonary infection, and inflammation, impairing pulmonary function [101, 102]. Pulmonary disease is the most serious manifestation of CF, which causes most of the morbidity and mortality in people with CF [100, 101]. As such, CF patients require lifelong follow‐up and imaging. Clinical assessments, conventional pulmonary function tests, and imaging such as chest CT are accepted standards for the long‐term follow‐up of CF [55]. Due to the concerns over repeated radiation exposure from chest CT scans for long‐term monitoring, recent studies have emphasized radiation‐free lung MRI as a viable alternative for assessing lung abnormalities in CF [3, 55, 103].

Lung MRI can assess the inflammatory and morphological changes in the lung while assessing regional and global changes in lung function. These parameters include ventilation homogeneity and percent defect calculated from MRI images for long‐term follow‐up, especially for pediatric patients [55, 103]. Automated quantification methods, such as quantifying the MRI T2 high‐signal‐intensity lung volume, can serve as imaging biomarkers for pathologic abnormalities in patients with CF [103]. The hyperpolarized gas MRI (such as ^3^He) is sensitivity to assess regional ventilation abnormalities of CF, while UTE oxygen‐enhanced MRI achieves comparable diagnostic performance to hyperpolarized ^3^He MRI [104]. More recently, Dohna et al. conducted a study that demonstrates the feasibility of PREFUL MRI for semiautomated quantitative assessment of perfusion and ventilation changes in assessing therapy response with CF [105]. The study on free‐breathing 3D UTE PREFUL MRI in 12 stable pediatric CF patients demonstrates the promising potential for UTE ventilation imaging in CF evaluation as a clinically feasible alternative to hyperpolarized ^129^Xe MRI for regional volumetric ventilation analysis [61]‌. Further multicenter studies with larger sample sizes are needed to validate and generalize that lung MRI can be part of the routine follow‐up of patients with CF.

### 3.5 Neonatal and Pediatric Lung Diseases

Neonatal and pediatric lung abnormalities, such as bronchopulmonary dysplasia, present unique manifestations from those found in adults. MRI without ionizing radiation can be a substitute for chest radiography or CT in certain situations. However, MRI imaging is technically challenged by small patient sizes, lower spatial resolution, and sensitivity to motion, especially for neonates [106, 107]. Additionally, neonatal and pediatric patients often struggle with breath‐holding during MRI examinations. While various MRI techniques for pediatric lung diseases, including those affecting neonates, have been researched, they have yet to be implemented in clinical practice [108, 109, 110].

UTE has been developed for neonatal lung MRI to calculate lung volumes, lung mass, and density, applying the initial feasibility of ^129^Xe lung MRI with bronchopulmonary dysplasia [109, 110, 111]. Another initial effort involves employing a deep learning model for neonatal pulmonary MRI segmentation, which enables the quantification of MRI features such as lung volume, surface area, shape, and intensity of bronchopulmonary dysplasia [112].

Additionally, functional MP, PREFUL MRI provides valuable information on ventilation and perfusion in children with large congenital diaphragmatic hernia, pediatric CF, and premature infants with bronchopulmonary dysplasia [64, 113, 114], which seems promising as a marker in the future. Structural and functional MRI techniques without exogenous contrast, such as PREFUL, have demonstrated the feasibility and promising potential in free‐breathing neonates and infants [64, 114, 115, 116]. PREFUL MRI does not require breath‐holds, and it correlates with hyperpolarized ^129^Xe MRI and pulmonary function tests in pediatric CF [64]. The VDP parameters in PREFUL MRI demonstrated high intravisit repeatability, but moderate intervisit repeatability in pediatric CF, suggesting their potential suitability as an outcome measure for future treatment response studies [116]. MRI perfusion measurements demonstrate correlations with spirometric lung function parameters and hold promise as radiation‐free follow‐up tools in adolescents after congenital diaphragmatic hernia [117]‌. In 2‐year‐old children after congenital diaphragmatic hernia, whole‐lung segmentation should be preferred over region‐of‐interest‐based approaches for MR lung perfusion quantification [118]. Although these findings are not yet clinically applicable, it is anticipated that these structural and quantitative MRI techniques, which utilize nonionizing radiation, will significantly enhance the diagnosis of pediatric lung diseases in the future.

## 4 Future Directions

In the future, the clinical application of pulmonary MRI will be propelled by technological optimization and clinical biomarkers validation, particularly driven by artificial intelligence (AI) advancements, ultimately enabling a shift from structural imaging to functional‐molecular precision diagnostics in pulmonary diseases. For technological optimization, free breathing with self‐gating will minimize motion artifacts and enhance patient comfort, particularly for pediatric or respiratory‐impaired populations. The UTE sequence or even ZTE sequence combined with functional imaging could simultaneously improve parenchymal signal intensity and enable quantitative perfusion analysis, offering sensitive biomarkers for early‐stage emphysema or fibrosis [59, 60, 61]. The transition from 2D to 3D volumetric lung imaging will optimize spatial resolution, enabling more accurate quantification of subtle pathological features and dynamic functional assessments.

AI technology, particularly deep learning algorithms, has revolutionized medical imaging workflows by enhancing image reconstruction quality and enabling advanced postprocessing capabilities, including accelerated acquisition protocols, signal standardization, and artifact reduction in dynamic imaging sequences [119, 120, 121, 122]. The primary strategy for accelerating MR imaging currently involves undersampling k‐space data during acquisition, particularly through deep learning‐based reconstruction frameworks that compensate for missing k‐space information. The undersampled MR image reconstruction techniques have evolved from supervised learning to semi‐supervised, and unsupervised learning approaches, with emerging federated learning approaches facilitating collaborative model training across multiple institutions while maintaining data privacy [119]. For hyperpolarized gas MRI, Zhou and colleagues first developed a cascaded convolutional neural networks model that integrates prior knowledge from ^1^H images to reconstruct hyperpolarized gas images from highly undersampled k‐space effectively. Their approach outperformed traditional undersampling methods, enhancing the application of deep learning in gas MRI reconstruction [120]. Additionally, a deep cascade of residual dense networks was also developed to accelerate high‐quality multiple *b* value gas diffusion‐weighted MRI for lung morphometry, improving patient tolerance by reducing acquisition time [121].

Meanwhile, clinically validated MRI biomarkers and AI‐assisted diagnosis of pulmonary MRI represent crucial future directions in enhancing diagnostic accuracy. MRI‐derived phenotypes can elucidate the pathophysiology and outcomes of lung disorders. However, multicenter studies are needed to validate these quantitative parameters against pulmonary function tests, histopathology, and prognosis, establishing standardized clinical thresholds. While AI‐assisted diagnosis of lung MRI remains a relatively underexplored research domain, emerging deep learning technologies demonstrate potential in enhancing MRI ventilation assessment via image synthesis and advanced image segmentation [123, 124, 125]. Capaldi and colleagues developed a convolutional neural network to create synthetic ventilation MRI scans from free‐breathing proton MRI‐specific ventilation maps, which achieved a 90% dice similarity coefficient for ventilated regions between ^3^He MRI and the deep learning‐generated ventilation MRI, with a strong correlation in ventilation defect percentages [123]. However, unimodal AI architectures exhibit inherent limitations in addressing cross‐domain complexity due to their susceptibility to modality. Consequently, a multimodal deep learning approach that combines ^129^Xe‐MRI and ^1^H‐MRI within a dual‐channel convolutional neural network was developed to evaluate ventilation defect percentage more accurately than single‐channel methods [124]. The heterogeneity introduced by discrepancies in MRI protocols and vendor‐specific systems poses a major obstacle to developing generalizable deep learning frameworks, resulting in compromised generalization capabilities across heterogeneous clinical data sets [125]. The prolonged timelines and substantial investments often required to navigate rigorous optimization, regulatory approvals, and scalability challenges hinder novel complex MRI sequences or AI‐driven methods from achieving clinical readiness and market viability. Therefore, to address the robustness of segmentation from variations in MR acquisition protocols or vendor, a generalizable deep learning‐based segmentation algorithm was developed to accurately delineate the lung cavity across diverse multicenter, multi‐vendor, and multi‐disease ^1^H‐MRI data sets [125].

Finally, portable and ultra‐low‐field MRI may enable bedside or home‐based lung monitoring for chronic disease management. The ultra‐low magnetic field strength (0.05, 0.2, or 0.55 T) MRI yields a lower MR signal but also fewer artifacts for lung imaging [8, 9, 10, 126, 127], which may have promising potential in lung imaging. Over the early years, ultra‐low field lung MRI has proven to be a fast and reliable no‐ionizing radiation technique as an alternative to the pediatric chest X‐ray of the lung [126]. For functional assessment of the lung at ultra‐low‐field strength MRI, ventilation maps and parameters can be generated by the signal changes between each image over the respiratory cycle, and regional lung function can be assessed by oxygen‐enhanced functional imaging [127]. Combining low field strength (0.55 T) with high‐performance imaging technology is also promising for further development. An initial study demonstrated that high‐performance low‐field‐strength (0.55 T) MRI can provide improved magnetic field homogeneity, resulting in reduced image distortion in the lungs and upper airway compared to a 1.5 T scanner [128]. Therefore, ultra‐low‐field lung MRI offers a safer and more cost‐effective imaging alternative, facilitating the more convenient diagnosis of pulmonary conditions while minimizing radiation exposure and healthcare costs.

## 5 Summary

The nonionizing radiation, combined with its ability to provide structural and regional functional information about the lung, makes lung MRI an attractive alternative to radiography‐based methods like CT, especially in patients who require longitudinal monitoring or are sensitive to radiation. However, several challenges still remain in the broader clinical adoption of MRI for lung diseases, such as further validating its diagnostic accuracy and establishing standardized protocols. Establishing standardized protocols across different MRI systems and institutions will ensure reproducibility and reliability of results, facilitating broader clinical adoption. Despite these hurdles, advancements in MRI hardware and imaging sequences, such as UTE and ZTE techniques, are pushing the field forward, particularly for the challenges posed by the inherent relatively short T2* of lung tissues. These improvements promise enhanced image quality and shorter scan times, potentially making MRI a more practical tool in routine clinical settings.

Integration of new technologies, such as AI for faster acquisitions and improved image quality, will help overcome the barriers, opening the door for MRI to play a more prominent role in lung disorder diagnosis and management. Additionally, the implementation of ultra‐low‐field MRI presents a promising avenue for lung disorders. As these innovations become more widely implemented, MRI is poised to complement and, in some cases, replace CT for certain pulmonary conditions, especially in cases where radiation exposure is a concern.
